# A causal effects of gut microbiota in the development of migraine

**DOI:** 10.1186/s10194-023-01609-x

**Published:** 2023-07-17

**Authors:** Qiang He, Wenjing Wang, Yang Xiong, Chuanyuan Tao, Lu Ma, Junpeng Ma, Chao You, The International Headache Genetics Consortium, Verneri Anttila, Ville Artto, Andrea C. Belin, Anna Bjornsdottir, Gyda Bjornsdottir, Dorret I. Boomsma, Sigrid Børte, Mona A. Chalmer, Daniel I. Chasman, Bru Cormand, Ester Cuenca-Leon, George Davey-Smith, Irene de Boer, Martin Dichgans, Tonu Esko, Tobias Freilinger, Padhraig Gormley, Lyn R. Griffiths, Eija Hämäläinen, Thomas F. Hansen, Aster V. E. Harder, Heidi Hautakangas, Marjo Hiekkala, Maria G. Hrafnsdottir, M. Arfan Ikram, Marjo-Riitta Järvelin, Risto Kajanne, Mikko Kallela, Jaakko Kaprio, Mari Kaunisto, Lisette J. A. Kogelman, Espen S. Kristoffersen, Christian Kubisch, Mitja Kurki, Tobias Kurth, Lenore Launer, Terho Lehtimäki, Davor Lessel, Lannie Ligthart, Sigurdur H. Magnusson, Rainer Malik, Bertram Müller-Myhsok, Carrie Northover, Dale R. Nyholt, Jes Olesen, Aarno Palotie, Priit Palta, Linda M. Pedersen, Nancy Pedersen, Matti Pirinen, Danielle Posthuma, Patricia Pozo-Rosich, Alice Pressman, Olli Raitakari, Caroline Ran, Gudrun R. Sigurdardottir, Hreinn Stefansson, Kari Stefansson, Olafur A. Sveinsson, Gisela M. Terwindt, Thorgeir E. Thorgeirsson, Arn M. J. M. van den Maagdenberg, Cornelia van Duijn, Maija Wessman, Bendik S. Winsvold, John-Anker Zwart

**Affiliations:** 1 Department of Neurosurgery, West China Hospital, Sichuan University, 37 Guoxue Lane, Wuhou District, Sichuan, Chengdu, 610041 China; 2 Department of Pharmacy, Institute of Metabolic Diseases and Pharmacotherapy, West China Hospital, Sichuan University, Chengdu, China; 3 Department of Urology, West China Hospital, Sichuan University, Chengdu, China; 4 Department of Neurosurgery, Bazhong People’s Hospital of Pingchang County, Bazhong, Sichuan China

**Keywords:** Gut microbiome, Migraine, Migraine with aura, Migraine without aura, Causal association, Mendelian randomization

## Abstract

**Background:**

The causal association between the gut microbiome and the development of migraine and its subtypes remains unclear.

**Methods:**

The single nucleotide polymorphisms concerning gut microbiome were retrieved from the gene-wide association study (GWAS) of the MiBioGen consortium. The summary statistics datasets of migraine, migraine with aura (MA), and migraine without aura (MO) were obtained from the GWAS meta-analysis of the International Headache Genetics Consortium (IHGC) and FinnGen consortium. Inverse variance weighting (IVW) was used as the primary method, complemented by sensitivity analyses for pleiotropy and increasing robustness.

**Results:**

In IHGC datasets, ten, five, and nine bacterial taxa were found to have a causal association with migraine, MA, and MO, respectively, (IVW, all *P* < 0.05). *Genus.Coprococcus3* and *genus.Anaerotruncus* were validated in FinnGen datasets. Nine, twelve, and seven bacterial entities were identified for migraine, MA, and MO, respectively. The causal association still exists in *family.Bifidobacteriaceae* and *order.Bifidobacteriales* for migraine and MO after FDR correction. The heterogeneity and pleiotropy analyses confirmed the robustness of IVW results.

**Conclusion:**

Our study demonstrates that gut microbiomes may exert causal effects on migraine, MA, and MO. We provide novel evidence for the dysfunction of the gut-brain axis on migraine. Future study is required to verify the relationship between gut microbiome and the risk of migraine and its subtypes and illustrate the underlying mechanism between them.

**Supplementary Information:**

The online version contains supplementary material available at 10.1186/s10194-023-01609-x.

## Introduction

Migraine has been recognized as the first leading cause of disability in adult populations less than 50 years [1], and approximately 14% of adults suffer from migraine [2]. The typical symptoms are photophobia, phonophobia, and cutaneous allodynia. Some symptoms of gastrointestinal tract (GI), including nausea, vomiting, and diarrhea, are frequently present in patients with migraine [3]. Although the exact pathological mechanism remains unclear, multiple factors are involved in the development of migraine, including gut-brain axis [4].

The gut-brain axis refers to the bidirectional association between the gut and brain. On the one hand, the brain normally regulates gut function by sensory and secreting hormonal factors. On the other hand, multiple factors in gut, including inflammatory mediators, gut microbiota profile, and stress hormones, affect the function of the central nervous system [3, 5]. The dysfunction of the gut-brain axis has been involved in several neurological diseases, such as multiple sclerosis, Alzheimer's disease, and migraine [3, 5]. As one of the major components in gut-brain axis, emerging evidence has suggested that dysbiosis of gut microbiota could affect migraine [6, 7]. In a study including 108 participants (54 cases and 54 matched controls) in elderly women, distinct differences in gut microbiota and function were detected between migraineurs and health controls [8]. The abundance of alpha diversity was decreased in elderly women with migraine. Meanwhile, the enrichment analysis of Kyoto Encyclopedia of Genes and Genomes orthologous levels. Firmicutes, especially Clostridium spp., were significantly increased in the migraine group. Bai and his colleague have found that the abundance of gut microbiota is significantly different between children with and without migraines [9]. Moreover, the incidence of a variety of GI disorders was higher in migraineurs than in the general population [10, 11]. Diarrhea, constipation, and gastroesophageal reflux are more frequent in patients with migraine. In addition, the alteration in gut microbiota has been observed in these GI disorders, the use of symbiotics in patients with migraine showed an improvement in the mean frequency [12]. Recently, however, in a systematic review of randomized placebo-controlled trials regarding probiotic supplements on the effect of migraine, results reported in nearly 70 trials were inconsistent- some claimed no significant change in migraine frequency and intensity, whileothers showed significant improvement [13]. Currently, these evidence suggests that we should put effort into research in gut-microbiome-migraine interaction to provide novel insights concerning migraine attack prevention and treatment [14].

However, information about whether and how the altered gut microbiota affects the development of migraine remains unknown. Gut microbiota constitutes a functional complex of the ecosystem, and it is still unclear whether one or multiple bacterial traits are involved in the development of migraine. Clinical studies, mostly observational ones in which the results are readily impacted by confounding factors, shave potential shortcomings such as limited sample size and retrospective design, impeding our understanding of this complex disease.

Mendelian randomization (MR) is a robust and effective method using genetic variants (single-nucleotide polymorphisms, SNPs) to explore the causal effects of gut microbiome on migraine [15]. Based on the random principle of meiosis, SNPs are assorted in the forming of a zygote during gestation randomly [12, 16], and the results of MR analyses are not susceptible to reverse causality and confusion. Previous studies have identified a causal relationship between gut microbiome and several neurological diseases using MR analysis, including stroke [17], Parkinson's Disease [18], and epilepsy [19], indicating the association of the gut-brain axis. The causal association between gut microbiota and migraine is lacking. Therefore, this study explored the possible causal association between each bacterial taxa and migraine through MR analyses, which may provide the theoretical basis for the gut-brain axis and provide novel insights for the prevention of migraine.

## Methods

### Data sources of gut microbiome

For gut microbiome, the summary-level datasets were retrieved from a large-scale gene-wide association study (GWAS) of the MiBioGen consortium [20]. The dataset includes a total of 18,340 samples of 16S rRNA gene sequencing data from 24 population-based cohorts. Most participants were of European ancestry (16 cohorts, 13,266 samples). A total of 211 gut microbiomes from genus to phylum level were identified. All bacterial traits were analyzed by three 16S rRNA regions and rarefied to 10,000 reads for rarefaction reproducibility, with 131 genera, 16 classes, 35 families, 20 orders, and 9 phyla being identified. Sex and age covariates were adjusted in all cohorts [20]. Detailed information on the gut microbiome was described in the original article [20] and was available on the website https://mibiogen.gcc.rug.nl.

### Data sources of migraine, MA and MO

The summary-level of datasets regarding migraine were collected from the GWAS meta-analysis of the International Headache Genetics Consortium (IHGC), which included European participants from 22 studies with 59,674 cases and 316,078 controls [21]. According to the diagnostic criteria from the International Headache Society, migraine was diagnosed using code G43 in the International Classification of Disease-10th revision [22]. The approaches of diagnosis covered self-report, questionnaires assessing diagnostic criteria, and diagnosis by a trained clinician interviewer. Two migraine subtypes, MA and MO, were included in our study. MA comprised 4,837 cases and 49,174 controls, and MO included 4,833 cases and 106,834 controls.

The other migraine dataset was obtained from the FinnGen study. Summary statistics data associated with migraine from R4 forms of GWAS [23]. Migraine was defined by code 346 in ICD-8 in the FinnGen consortium. A total of 10,536 migraine cases and 208,845 controls were included. Two prevalent forms of migraine were also included: MA (6,332 cases and 144,883 controls) and MO (8,348 cases and 139,622 controls).

### Ethical approvement

All summary-level datasets in our study were retracted from de-identified public data/studies. Ethical approval and informed consent were obtained by the ethics committee previously. Ethical approval was thus exempted from our study.

### Genetic instrument selection

Considering the small number of IVs obtained, the genetic instruments associated with bacterial traits were selected at locus-wide significance level (*P* < 1 × 10^–5^). The independent SNPs were obtained with the threshold of an r^2^ < 0.01 and clumping window (10,000 kb), using the European population as a reference. The instrument variables (IVs) were shown in Table S1. Furthermore, MR Pleiotropy RESidual Sum and Outlier (MR-PRESSO) approach was utilized to explore significant SNPs accounting for possible pleiotropy [24], and the outlier SNPs were removed. The results of F-statistics = (Bets/Se) [2] represent the strength of MR, and SNP with the value of F-statistics < 10, indicative of insufficient strength [25], was abandoned. In this formula, beta is the correlation coefficient between SNPs and traits (bacterial trait and IA). All value of F-statistics exceeds 10 in this MR study. In addition, we also set the *P* at the threshold of < 1e-8 to screen SNPs at a revised genome-wide significance threshold. The IVs for IHGC and FinnGen were shown in Table S2 and Table S3, respectively.

### Main statistical analyses

The random effects inverse variance weighting (re-IVW) approach was the primary method to explore the causal associations in the MR study since this analysis can provide a robust causal estimate in the absence of directional pleiotropy (no violation of the independence assumption). *P* < 0.05 represents statistical significance. The false discovery rate (FDR) was introduced to adjust the results in multiple comparisons (Benjaminiand Hochberg). All analyses were conducted using “TwoSampleMR”, “mr.raps”, “MRPRESSO”, “frostplot” and “ggplot2” in the R software (version 4.2.0, The R Foundation, Vienna, Austria).

### Sensitivity analyses

Multiple methods including MR-Egger, Weight median, Maximum likelihood, MR robust adjusted profile score (MR-RAPS), and MR-PRESSO were performed to examine the causal association between gut microbiome and migraine in sensitivity analyses. On the assumption of instrument strength independent of direct effect (InSIDE), MR-Egger could evaluate the existence of pleiotropy with the intercept term. When the intercept term is close to zero, horizontal pleiotropy does not exist and the results of both IVW and MR-Egger are similar [26]. Weighted median-based MR analysis is also used to correct the estimation of the causal effect, assuming that at least half of the IVs are invalid [27, 28]. Similar to IVW, the assumption of Maximum likelihood is the absence of heterogeneity and horizontal pleiotropy. If the hypothesis is true, the findings of Maximum likelihood are unbiased. In addition, the standard errors are smaller than IVW [29]. Significant outliers in MR-PRESSO analysis are removed to reduce horizontal pleiotropy. The validity of MR-PRESSO requires up to 50% of valid instruments and depends on InSIDE assumption [24]. MR-RAPS analysis was performed to verify the robustness of our conclusion. When weak SNPs exist, MR-RAPS analysis can provide higher statistical power [30]. Cochran's *Q* statistic was used to explore the heterogeneity among variant-specific estimates. In addition, leave-one-out analysis was performed to verify the robustness of the conclusion.

### Reverse MR analysis of the causal effects of migraine, MA and MO on gut microbiome

To examine the causal effects of genetically predicted migraine, MA, and MO on gut microbiome, we collected the IVs for migraine, MA, and MO at the threshold of *P* < 1e-5 (Tables S4-S9) and *P* < 1e-8 (Tables S10-S11). The statistical methods used in reverse MR analyses have been described before.

## Results

### Genetic instrument variables for gut microbiome

**Fig. 1:**
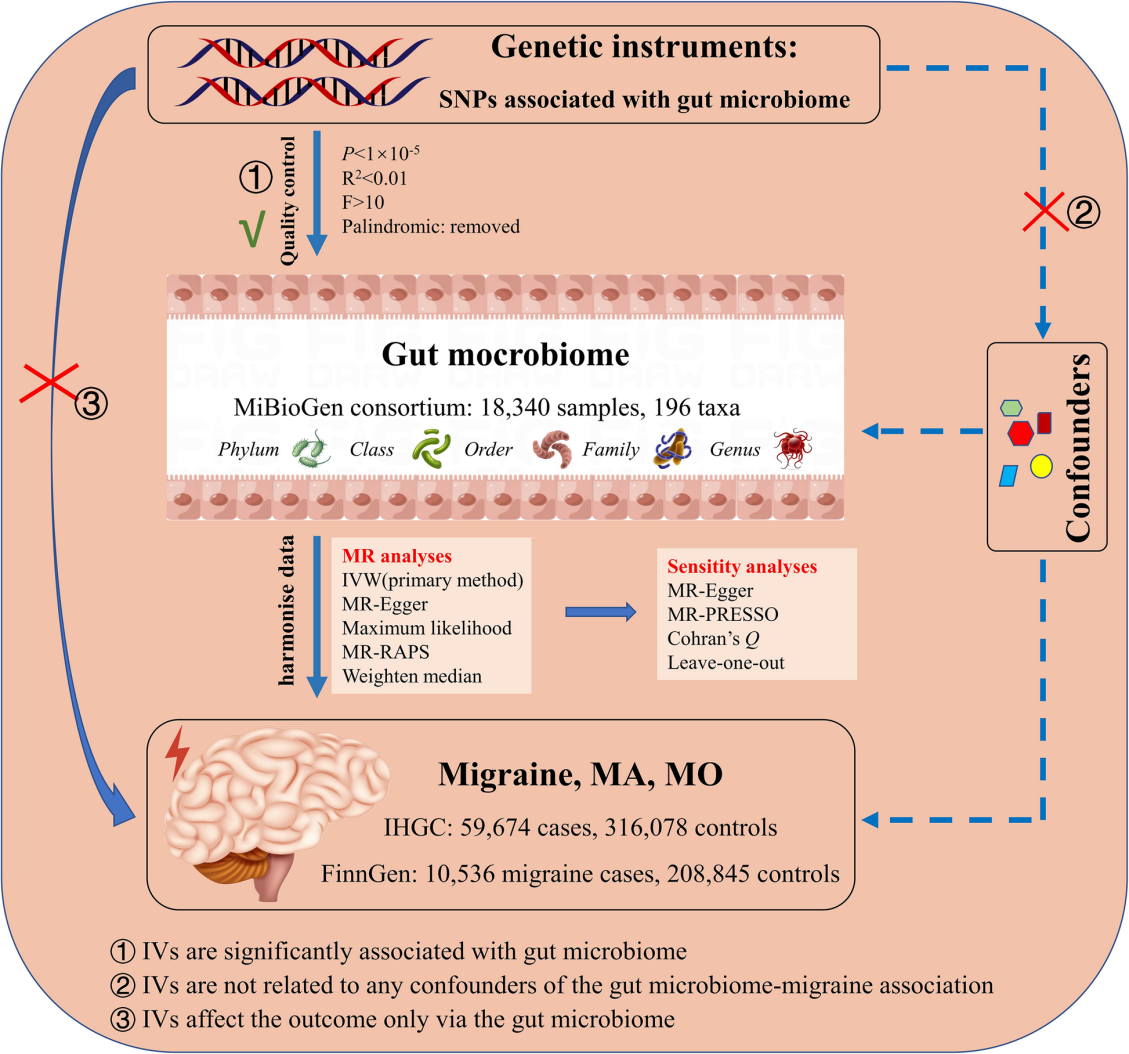
Study design of the two-sample Mendelian randomization for the effect of the genetically predicted gut microbiome on migraine, MA, and MO. SNP, single nucleotide polymorphism; MA, migraine with aura; MO, migraine without aura; IHGC, international headache genetics consortium; IV, instrumental variables; IVW, IVW, inverse variance weighted; RAPS, robust adjusted profile score; MR-PRESSO, MR Pleiotropy RESidual Sum and Outlier

The flow chart of this study was shown in Fig. 1. A total of 211 bacterial traits including 5 biological levels (phylum, class, order, family, and genus) were collected in our study. Fifteen bacterial traits were removed due to unknown traits. Collectively, a total of 196 bacterial traits were included in MR analyses for migraine, MA, and MO in IHGC datasets and FinnGen datasets. Positive MR results of causal effects of gut microbiome on migraine, MA, and MO in IHGC datasets were shown in Table 1. According to the results of IVW, ten, five, and nines bacterial traits showed a causal association between gut microbiome and migraine, MA, and MO in IHGC datasets (Table 1).

**Table 1:** Positive MR results of causal links between gut microbiome and migraine, migraine with aura and migraine without aura in the IHGC GWAS datasets at the threshold of *P* < 1e-5 in MR analysis

**Outcome**	**Exposure**	**Power**	**F-statistics**	**Method**	**No. SNP**	***Pval***	**OR**
**Migraine**	**Class**						
	Actinobacteria	0.89	25.78	IVW	16	0.0141	1.13(1.03–1.25)
	**Family**						
	ClostridialesvadinBB60group	0.50	22.03		15	0.0445	1.09(1.00–1.18)
	**Genus**						
	Eubacteriumnodatumgroup	0.30	21.58	IVW	11	0.0126	0.92(0.87–0.98)
	Eubacteriumrectalegroup	0.75	21.22	IVW	9	0.0483	0.86(0.75–1.00)
	Ruminococcusgnavusgroup	0.40	21.90		12	0.0402	0.91(0.83–1.00)
	Coprobacter	0.47	21.19	IVW	11	0.0347	1.09(1.01–1.17)
	LachnospiraceaeUCG001	0.96	22.21	IVW	12	0.0006	1.18(1.07–1.30)
	LachnospiraceaeUCG008	0.76	23.11	IVW	11	0.0014	1.14(1.05–1.23)
	Marvinbryantia	0.97	22.02		10	0.0028	1.21(1.07–1.36)
	Roseburia	0.89	20.84	IVW	14	0.0234	1.15(1.02–1.30)
**MA**	**Genus**						
	Coprococcus3	0.77	21.52	IVW	8	0.0490	1.35(1.00–1.82)
	LachnospiraceaeUCG008	0.44	23.11	IVW	11	0.0317	1.19(1.02–1.40)
	Marvinbryantia	0.99	22.02		10	0.0005	1.48(1.19–1.84)
	RuminococcaceaeNK4A214group	0.88	21.50	IVW	14	0.0051	0.72(0.57–0.91)
	**Order**						
	MollicutesRF9	0.95	20.50	IVW	13	0.0009	1.35(1.13–1.61)
**MO**	**Class**						
	Actinobacteria	0.93	26.16	IVW	15	0.0142	1.25(1.05–1.50)
	Melainabacteria	0.31	22.05		10	0.0410	0.87(0.76–0.99)
	**Family**						
	BacteroidalesS24.7group	0.36	23.14	IVW	8	0.0491	0.84(0.70–1.00)
	FamilyXI	0.16	22.42	IVW	8	0.0480	0.89(0.80–1.00)
	**Genus**						
	Eubacteriumfissicatenagroup	0.27	21.16	IVW	9	0.0283	1.15(1.02–1.31)
	Eubacteriumnodatumgroup	0.21	21.58	IVW	11	0.0218	0.89(0.80–0.98)
	Anaerotruncus	0.91	20.84	IVW	13	0.0496	1.28(1.00–1.63)
	Catenibacterium	0.21	21.23		5	0.0386	1.17(1.01–1.37)
	Parasutterella	0.54	22.29	IVW	15	0.0333	0.84(0.71–0.99)

*MR* Mendelian randomization, *MA* Migraine with aura, *MO* Migraine without aura, *IHGC* International headache genetics consortium, *GWAS* Genome-wide association study, *OR* Odds ratio, *CI* Confidential interval

### Causal effects of the genetically predicted gut microbiome on migraine, MA, MO at the threshold of *P* < 1e-5 in IHGC datasets in MR analyses

**Fig. 2:**
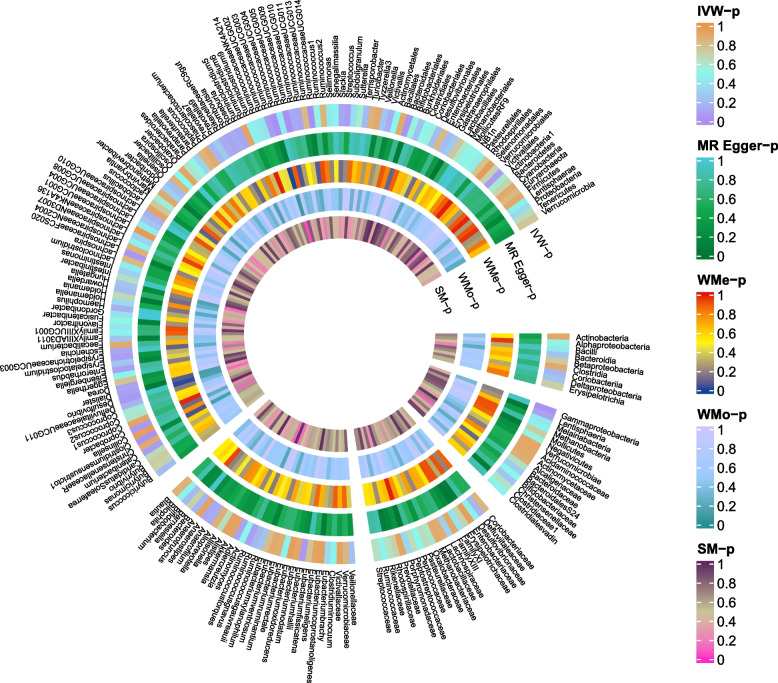
Causal effect of the gut microbiome on migraine in IHGC datasets based on MR analyses. From outside to inside, the P values of IVW, MR Egger, WMe, WMo, and SM are represented, respectively. IVW, inverse variance weighted; WMe, weighted median; WMo, weighted mode; SM, simple mode. MR, mendelian randomization; IHGC, international headache genetics consortium

**Fig. 3:**
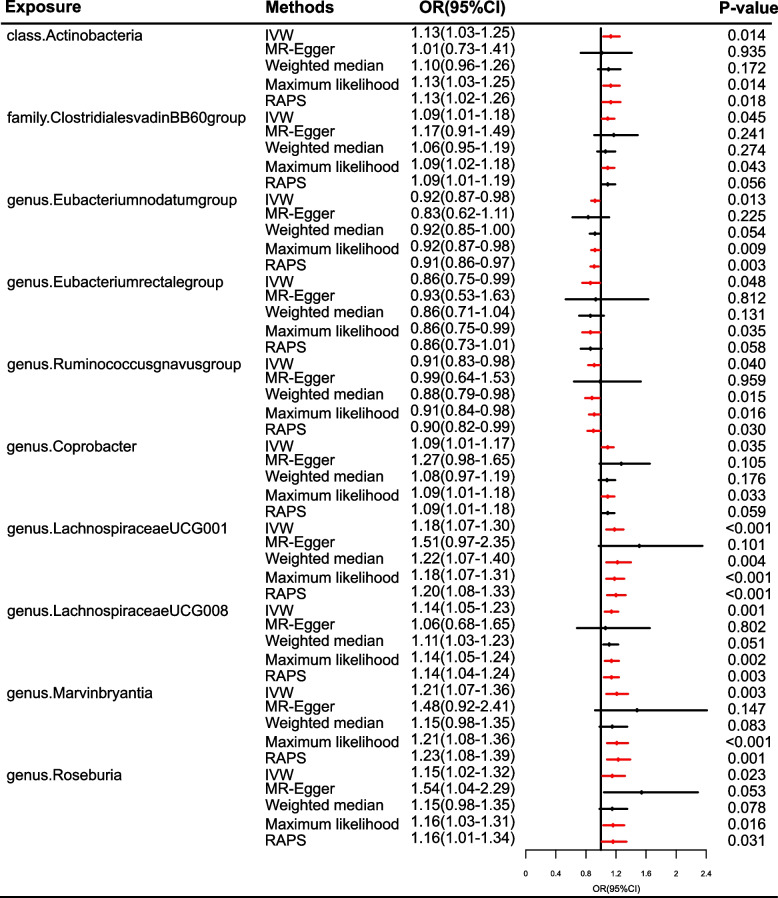
Causal effect estimates of the gut microbiome on migraine in IHGC datasets. OR, odds ratio; CI, confidence interval; IVW, inverse variance weighted method; RAPS, robust adjusted profile score; MR, mendelian randomization; IHGC, international headache genetics consortium

**Fig. 4:**
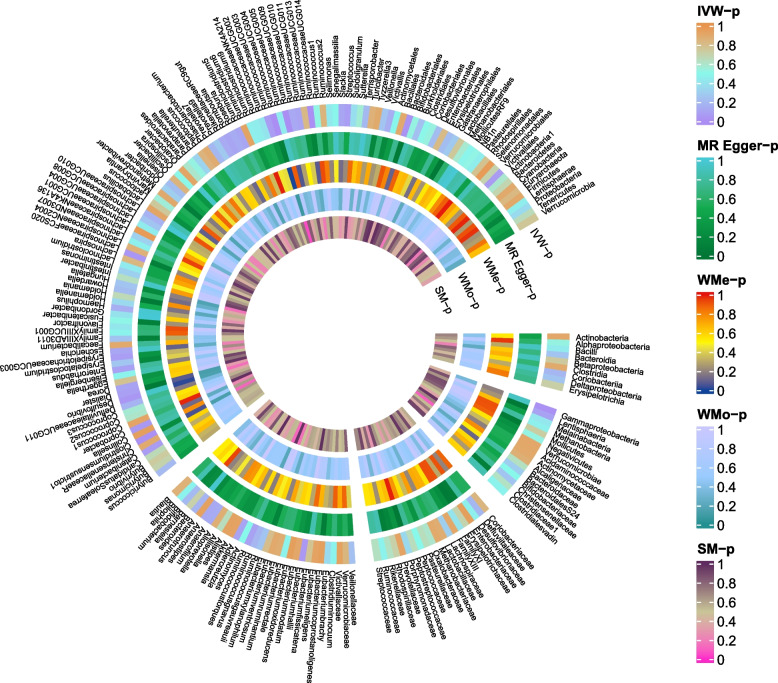
Causal effect of the gut microbiome on MA in IHGC datasets based on MR analyses. From outside to inside, the P values of IVW, MR Egger, WMe, WMo, and SM are represented, respectively. MA, migraine with aura; IVW, inverse variance weighted; WMe, weighted median; WMo, weighted mode; SM, simple mode. MR, mendelian randomization; IHGC, international headache genetics consortium

The causal effects of 196 bacterial taxa on migraine, MA, and MO risk were shown in Figs. 1, 2, 4, and 6, respectively. As shown in Fig. 2, Table S12, and Fig. 3, genetically predicted *class.Actinobacteria* (*P* = 0.014), *family.ClostridialesvadinBB60group* (*P* = 0.045), *genus.Coprobacter* (*P* = 0.035), *genus.LachnospiraceaeUCG001* (*P* < 0.001), *genus.LachnospiraceaeUCG008* (*P* = 0.001), *genus.Marvinbryantia* (*P* = 0.003), *genus.Roseburia* (*P* = 0.023) were causally related to the increased risk of migraine. The odds ratios (ORs) for these links were 1.13(95% confidential interval [CI] = 1.03–1.25) for *class.Actinobacteria*, 1.09(95%CI = 1.01–1.18) for *family.ClostridialesvadinBB60group*, 1.09(95%CI = 1.01–1.17) for *genus.Coprobacter*, 1.18(95%CI = 1.07–1.30) *genus.LachnospiraceaeUCG001*, 1.14 (95%CI = 1.05–1.23) for *genus.LachnospiraceaeUCG008*, 1.21(95%CI = 1.07–1.36) for *genus.Marvinbryantia*, 1.54(95%CI = 1.02–1.32) for *genus.Roseburia*. In contrast, inverse causal association between *genus.Eubacteriumnodatumgroup* (*P* < 0.013), *genus.Eubacteriumrectalegroup* (*P* < 0.048), *genus.Ruminococcusgnavusgroup* (*P* < 0.040) and migraine were observed. The ORs for these inverse associations were 0.92(95%CI = 0.87–0.98) for *genus.Eubacteriumnodatumgroup*, 0.86(95%CI = 0.75–0.99) for *genus.Eubacteriumrectalegroup*, 0.91(95%CI = 0.83–0.98) for *genus.Ruminococcusgnavusgroup* (Fig. 3). A similar trend was observed in maximum likelihood analyses. However, no bacterial traits passed the FDR correction. There was limited power (< 0.8) to test the causality of these bacterial traits on migraine.

**Fig. 5:**
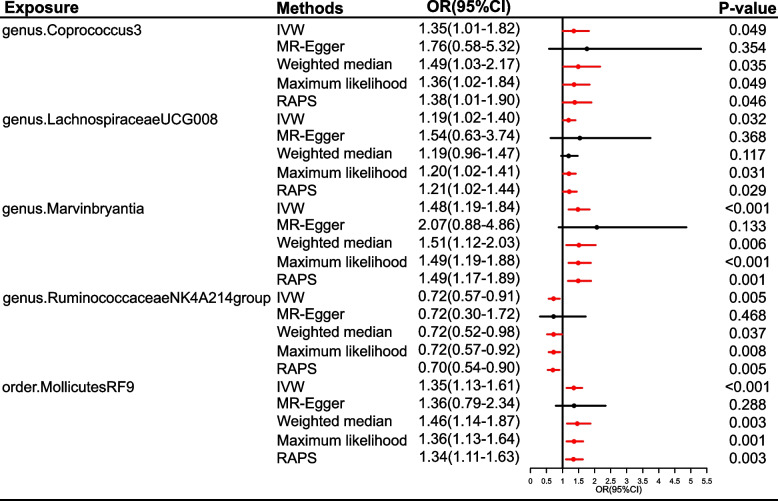
Causal effect estimates of the gut microbiome on MA in IHGC datasets. OR, odds ratio; CI, confidence interval; IVW, inverse variance weighted method; RAPS, robust adjusted profile score; MR, mendelian randomization; IHGC, international headache genetics consortium; MA, migraine with aura

The causal effects of 196 gut microbiomes on MA in IHGC datasets were shown in Fig. 4 and Table S13. A total of five bacterial features were causally related to the MA risk (Fig. 5). Among these bacterial features, MA risk was intensified by *genus.Coprococcus3* (OR = 1.35, 95%CI = 1.01–1.82, *P* = 0.049), *genus.LachnospiraceaeUCG008* (OR = 1.19, 95%CI = 1.02–1.40, *P* = 0.032), *genus.Marvinbryantia* (OR = 1.48, 95%CI = 1.19–1.84, *P* < 0.001), and *order.MollicutesRF9* (OR = 1.35, 95%CI = 1.13–1.61, *P* < 0.001), while *genus.RuminococcaceaeNK4A214group* decreased the risk of MA (OR = 0.72, 95%CI = 0.57–0.91, *P* = 0.005, Fig. 5). A similar trend was detected in the RAPS and maximum likelihood analyses. All bacterial traits failed to pass the FDR correction. There was limited power (< 0.8) to test the causality of these bacterial traits on MA.

**Fig. 6:**
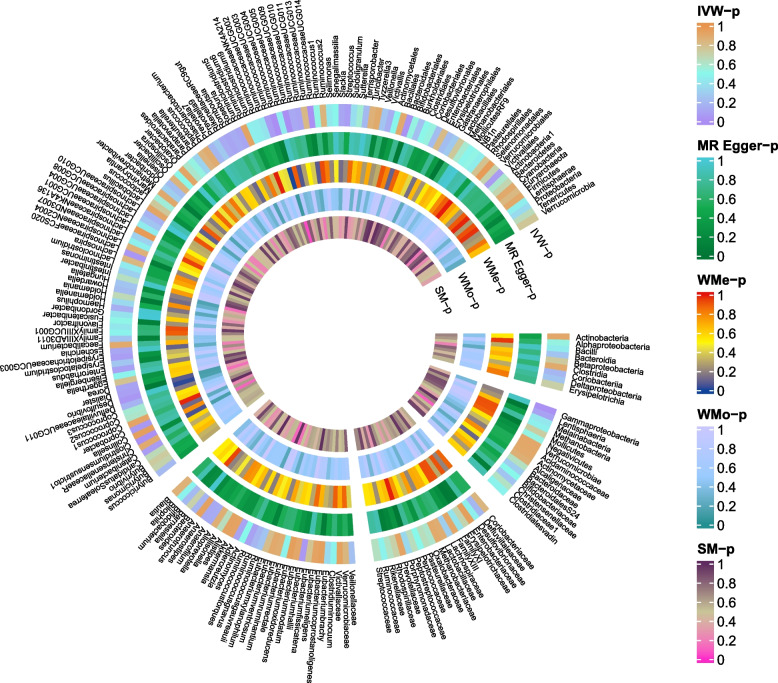
Causal effect of the gut microbiome on MO in IHGC datasets based on MR analyses. From outside to inside, the P values of IVW, MR Egger, WMe, WMo, and SM are represented, respectively. MO, migraine without aura; IVW, inverse variance weighted; WMe, weighted median; WMo, weighted mode; SM, simple mode. MR, mendelian randomization; IHGC, international headache genetics consortium

**Fig. 7:**
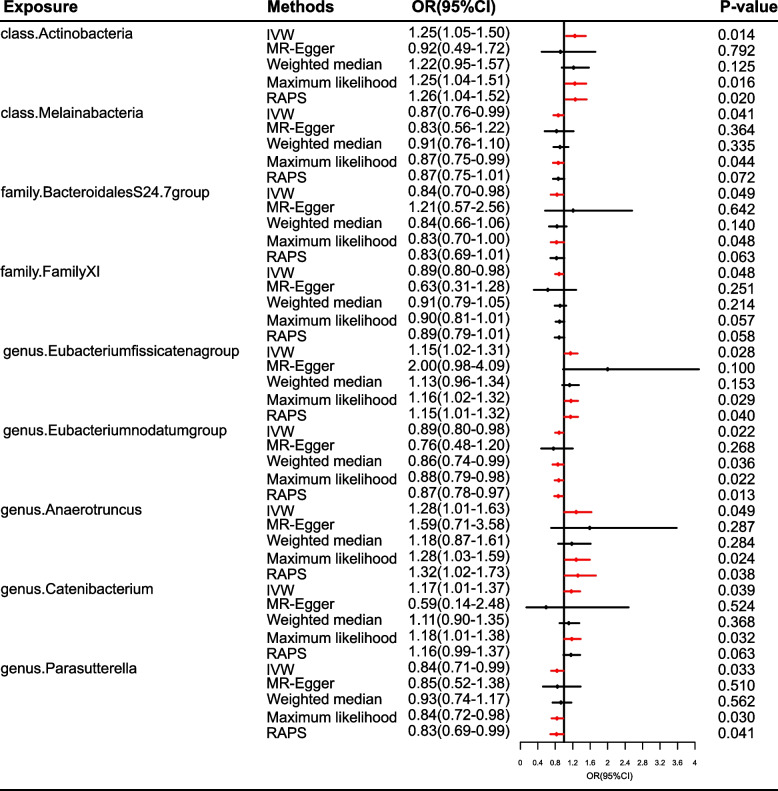
Causal effect estimates of the gut microbiome on MO in IHGC datasets. OR, odds ratio; CI, confidence interval; IVW, inverse variance weighted method; RAPS, robust adjusted profile score; MR, mendelian randomization; IHGC, international headache genetics consortium; MO, migraine without aura

Figure 6 and Table S14 did show the causal effects of 196 gut microbiomes on MO. Figure 7 displayed these causal links. Genetically predicted *class.Actinobacteria* (OR = 1.25, 95%CI = 1.05–1.50, *P* = 0.014), *genus.Eubacteriumfissicatenagroup* (OR = 1.15, 95%CI = 1.02–1.31, *P* = 0.028), *genus.Anaerotruncus* (OR = 1.28, 95%CI = 1.01–1.63, *P* = 0.049), and *genus.Catenibacterium* (OR = 1.17, 95%CI = 1.01–1.37, *P* = 0.039) were causally related to the increased risk of MO, while the risk of MO was decreased by *class.Melainabacteria* (OR = 0.87, 95%CI = 0.76–0.99, *P* = 0.041), *family.BacteroidalesS24.7group* (OR = 0.84, 95%CI = 0.70–0.98, *P* = 0.049), *family.FamilyXI* (OR = 0.89, 95%CI = 0.80–0.98, *P* = 0.048), *genus.Eubacteriumnodatumgroup* (OR = 0.89, 95%CI = 0.80–0.98, *P* = 0.022), and *genus.Parasutterella* (OR = 0.84, 95%CI = 0.71–0.99, *P* = 0.033, Fig. 7). All the IVW results of bacterial features failed to pass FDR correction (FDR > 0.05). In this part, no bacterial traits passed the FDR correction. There was limited power (< 0.8) to test the causality of these bacterial traits on MO.

In sensitivity analyses, leave-one-out analyses showed no significant SNPs for migraine, MA, and MO in IHGC datasets (Figures S1-S3). The results of MR-Egger and MR-PRESSO analyses demonstrated no signs of pleiotropy (Table S15). Moreover, the results of Cochran’s *Q* test demonstrated no signs of heterogeneity (Table S15).

*Genus.LachnospiraceaeUCG008* and *genus.Marvinbryantia* were common bacterial taxa between migraine and MA, and *class.Actinobacteria* and *genus.Eubacteriumnodatumgroup* were common bacterial taxa between migraine and MO (Figure S4).

### Causal effects of the genetically predicted gut microbiome on migraine, MA, MO at the threshold of *P* < 1e-5 in FinnGen datasets in MR analyses

The causal effects of 196 bacterial taxa on the risk of migraine, MA, and MO were shown in Figure S5. Positive results of their associations were shown in Table S16. Nine, twelve, and seven bacterial features were causally associated with the migraine, MA, and MO risk, respectively.

For migraine, the causal effects of 196 bacterial taxa on the risk of migraine were shown in Figure S5A and Table S17. Specifically, the risk of migraine was increased by *family.Actinomycetaceae* (OR = 1.17, 95%CI = 1.01–1.36, *P* = 0.047) and *order.Actinomycetales* (OR = 1.17, 95%CI = 1.01–1.36, *P* = 0.047) (Figure S6). In contrast, migraine risk was decreased by *class.Clostridia* (OR = 0.84, 95%CI = 0.72–0.98, *P* = 0.030), *family.Bifidobacteriaceae* (OR = 0.72, 95%CI = 0.65–0.81, *P* < 0.001), *genus.Bifidobacterium* (OR = 0.83, 95%CI = 0.72–0.96, *P* = 0.013), *genus.LachnospiraceaeNK4A136group* (OR = 0.89, 95%CI = 0.80–0.99, *P* = 0.035), genus.Olsenella (OR = 0.92, 95%CI = 0.86–0.99, *P* = 0.028), *order.Bifidobacteriales* (OR = 0.72, 95%CI = 0.65–0.81, *P* < 0.001), *order.NB1n* (OR = 0.92, 95%CI = 0.86–0.99, *P* = 0.044, Figure S6). A similar trend was observed in maximum likelihood analyses. The MR results of *family.Bifidobacteriaceae* and *order.Bifidobacteriales* passed the FDR correction (1.03E-06, 1.03E-06, respectively). The power (more than 0.8) was enough to explain the causality of *family.Bifidobacteriaceae* and *order.Bifidobacteriales* on migraine.

For MA, the causal effects of 196 bacterial taxa on the risk of MA were shown in Figure S5B and Table S18. *Genus.Coprococcus3* (*P* = 0.034), *genus.Oxalobacter* (*P* = 0.030), *genus.Phascolarctobacterium* (*P* = 0.046), *genus.Prevotella7* (*P* = 0.047), and *genus.RuminococcaceaeUCG003* (*P* = 0.037) were causally related to the increased risk of MA. The ORs for these traits were 1.29(95%CI = 1.02–1.63) for *genus.Coprococcus3*, 1.13(95%CI = 1.01–1.26) for *genus.Oxalobacter*, 1.22(95%CI = 1.01–1.49) *genus.Phascolarctobacterium*, 1.11(95%CI = 1.01–1.24) for *genus.Prevotella7*, and 1.22(95%CI = 1.01–1.46) for *genus.RuminococcaceaeUCG003* (Figure S7). Nevertheless, *family.BacteroidalesS24.7group* (OR = 0.84, 95%CI = 0.71–0.98, *P* = 0.048), *family.Bifidobacteriaceae* (OR = 0.77, 95%CI = 0.64–0.92, *P* = 0.004), *family.Clostridiaceae1* (OR = 0.78, 95%CI = 0.64–0.96, *P* = 0.022), *genus.ChristensenellaceaeR.7group* (OR = 0.73, 95%CI = 0.55–0.97, *P* = 0.029), *genus.Clostridiumsensustricto1* (OR = 0.79, 95%CI = 0.63–0.98, *P* = 0.029), *genus.Prevotella9* (OR = 0.82, 95%CI = 0.72–0.94, *P* = 0.004), and *order.Bifidobacteriales* (OR = 0.77, 95%CI = 0.64–0.92, *P* = 0.004) increased the risk of MA (Figure S7). Maximum likelihood analyses revealed a similar trend. However, no bacterial traits in this MR analyses passed the FDR correction (*P* > 0.05). There was limited power (< 0.8) to test the causality of these bacterial traits on MA.

As to MO, the causal effects of 196 bacterial taxa on the risk of MO were shown in Figure S5C and Table S19. The risk was increased by *genus.Anaerotruncus* (OR = 1.34, 95%CI = 1.04–1.71, *P* = 0.022), *genus.LachnospiraceaeUCG008* (OR = 1.17, 95%CI = 1.01–1.37, *P* = 0.041), and *genus.RuminococcaceaeUCG009* (OR = 1.17, 95%CI = 1.01–1.37, *P* = 0.039) (Figure S8). However, the risk of MO was decreased by *family.Bifidobacteriaceae* (OR = 0.73, 95%CI = 0.61–0.87, *P* < 0.001), *genus.Bifidobacterium* (OR = 0.80, 95%CI = 0.67–0.96, *P* = 0.015), *genus.Butyricicoccus* (OR = 0.76, 95%CI = 0.60–0.98, *P* = 0.034), *order.Bifidobacteriales* (OR = 0.73, 95%CI = 0.61–0.87, *P* < 0.001, (Figure S8). Both RAPS and maximum likelihood analyses demonstrated a similar trend. The IVW results of *family.Bifidobacteriaceae* and *order.Bifidobacteriales* passed the FDR correction (*P* < 0.05). The value of power tend to support causality of *family.Bifidobacteriaceae* and *order.Bifidobacteriales* on MO (power = 0.78).

No significant SNPs were detected in the leave-one-out analyses for migraine, MA, and MO in IHGC datasets (Figures S9-S11). The results of MR-Egger and MR-PRESSO analyses demonstrated no signs of pleiotropy (Table S20). Moreover, the results of Cochran’s *Q* test demonstrated no signs of heterogeneity (Table S20).

*Genus.Bifidobacterium* were common bacterial taxa between migraine and MO, and *family.Bifidobacteriaceae* and *order.Bifidobacteriales* were common bacterial taxa among migraine, MA, and MO (Figure S12).

### Common bacterial taxa between IHGC datasets and FinnGen datasets regarding migraine, MA, and MO at the threshold of *P* < 1e-5 in MR analyses

**Fig. 8:**
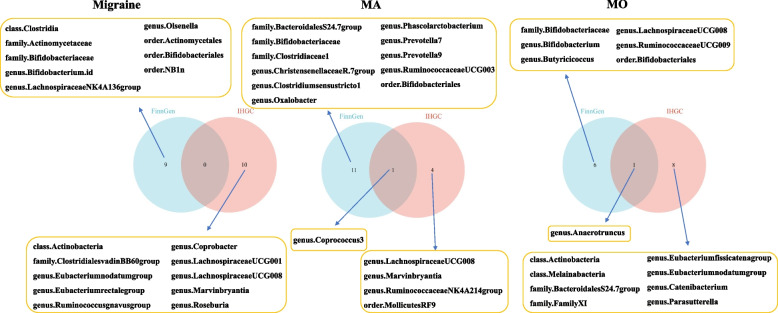
The common bacterial traits in IHGC datasets and FinnGen datasets for migraine, MA, and MO. IHGC, international headache genetics consortium; MA, migraine with aura; MO, migraine without aura

As shown in Fig. 8, no common bacterial trait was identified for migraine. However, *genus.Coprococcus3* was the common bacterial feature of MA between IHGC datasets and FinnGen datasets, and *genus.Anaerotruncus* was the common bacterial feature between the two datasets. The summarized results of the meta-analysis shown stable results for *genus.Coprococcus3* (OR = 1.31, 95%CI = 1.09–1.58) and *genus.Anaerotruncus* (OR = 1.31, 95%CI = 1.10–1.56).

### Causal effects of the genetically predicted gut microbiome on migraine, MA, MO in IHGC datasets and FinnGen datasets at the threshold of *P* < 1e-8 in MR analyses

As shown in the Table S21, *class.Actinobacteria* was not the risk factors for migraine and MO (*P* > 0.05) at the threshold of *P* < 1e-8 in IIHGC GWAS datasets. In FinnGen datasets (Table S22), *family.Bifidobacteriaceae* and *order.Bifidobacteriales* were common bacterial traits for migraine, MA, and MO. *Genus.Bifidobacterium* was common bacterial traits between migraine and MO. Genetically predicted *family.Bifidobacteriaceae* decreased the risk of migraine (Wald ratio: OR = 0.65, 95%CI = 0.52–0.81, *P* = 0.0001), MA (Wald ratio: OR = 0.57, 95%CI = 0.41–0.79, *P* = 0.0010), and MO (Wald ratio: OR = 0.68, 95%CI = 0.47–0.97, *P* = 0.0338). *Order.Bifidobacteriales* decreased the risk of migraine (Wald ratio: OR = 0.65, 95%CI = 0.52–0.81, *P* = 0.0001), MA (Wald ratio: OR = 0.57, 95%CI = 0.41–0.79, *P* = 0.0010), and MO (Wald ratio: OR = 0.68, 95%CI = 0.47–0.97, *P* = 0.0338). *Genus.Bifidobacterium* also decreased the risk of migraine (Wald ratio: OR = 0.66, 95%CI = 0.53–0.81, *P* = 0.0001) and MO (Wald ratio: OR = 0.68, 95%CI = 0.47–0.97, *P* = 0.0338).

### Causal effects of the genetically predicted migraine, MA, MO on gut microbiome at the threshold of *P* < 1e-5 in FinnGen and IHGC datasets in reverse MR analyses

As shown in the Figures S13-S15, no causal association between migraine, MA, MO and gut microbiome in IHGC datasets. The MR-Egger did not detect pleiotropy (Table S23). The leave-one-out analysis did not detect significant outliers (Figures S16-S18). In FinnGen datasets, no causal association between migraine, MO and gut microbiome was detected (Figures S19-S21). No pleiotropy was detected in MR-Egger analyses (Table S24). In Cochran’s *Q* analyses, no heterogeneity was observed (Table S24). The leave-one-out analyses did not detect any significant outliers (Figures S22-S24).

### Causal effects of the genetically predicted migraine, MA, MO on gut microbiome at the threshold of *P* < 1e-8 in FinnGen and IHGC datasets in reverse MR analyses

In the reverse analyses at the threshold of *P* < 1e-8, the causal association of migraine, MA, MO on gut microbiome on gut microbiome was not detected in IHGC datasets (Figure S25) and FinnGen datasets (Figure S26). No pleiotropy and no heterogeneity were detected using MR-Egger and Cochran’s *Q* test (Table S25). The leave-one-out analyses did not detect any significant outliers (Figures S27-S28). No SNP was collected in FinnGen datasets (Table S26).

## Discussion

In this MR study, we first identified the causal effect of bacterial entities in the gut microbiome on the risk of migraine, MA, and MO through multiple datasets. Concerning the IHGC datasets, our results demonstrated that ten, five, and nine bacterial taxa were found to have a causal association with migraine, MA, and MO, respectively. However, no bacterial traits pass the FDR correction. *Genus.Coprococcus3* and *genus.Anaerotruncus* were validated in FinnGen datasets. Nine, twelve, and seven bacterial entities were identified for migraine, MA, and MO, respectively. Both *family.Bifidobacteriaceae* and *order.Bifidobacteriales* were associated with the decreased risk of migraine, MA, and MO, and the association still existed after FDR correction. *Genus.Coprococcus3* and *genus.Anaerotruncus* were common traits in IHGC and FinnGen for MA and MO. In the MR analysis at the threshold of *P* < 1e-8, the causal association between *family.Bifidobacteriaceae*, *order.Bifidobacteriales* and migraine, MA, and MO remain stable. In reverse MR analysis, no causal association were identified. Collectively, our results may provide novel clues to illustrate the effect of specific bacterial features on the development of migraine and its subtypes. The regulation of specific bacterial traits may be helpful in migraine prevention and treatment.

The dysbiosis of gut microbiota in patients with migraine has been observed in both adults [8, 31, 32] and children [9]. A metagenome-wide association study performed in elderly women showed that unfriendly bacterial traits, such as Firmicutes levels Clostridium spp., were significantly increased in patients with migraine. Conversely, *Faecalibacterium prausnitzii*, *Bifidobacterium adolescentis*, and *Methanobrevibacter smithii* were detected in healthy controls [8]. In a cohort of 381 children (40 with migraine, 341 without migraine) aged 7 ~ 18 years, a distinct abundance of bacterial traits were detected between children with migraine and healthy controls. Higher abundances in genus of phylum *Bacteroidetes* (*Bacteroides*, *Parabacteroides*, and *Odoribacter*), *Actinobacteria* (*Eggerthella*, *Varibaculum*), *Firmicutes* (*SMB53*, *Lachnospira*, *Dorea*, *Veillonella*, *Anaerotruncus*, *Butyricicoccus*, *Coprobacillus*, *Eubacterium*), and *Proteobacteria* (*Sutterella*) were detected in children with migraine than in children without migraines [9]. In young females, the severity and duration of MA were positively related to dysbiosis [33]. Headache may also lead to the occurrence of dysbiosis. Miao and his colleagues have reported that headache due to meningitis could lead to dysbiosis in gut, and they think that inflammatory dural stimulation-induced cephalic headahce causes the alterations of gut microbiota profile and microbial metabolic pathways [34]. However, these studies cannot provide the causal association between dysbiosis of gut microbiota and migraine. In addition, these studies couldn't illustrate the role of the specific bacteria. In our MR study, our results demonstrated that Bifidobacterium and Actinobacteria for migraine and MO and *Anaerotruncus* for MO are risk factors, and *Butyricicoccus* may be a protective factor for MO. However, we did not identify the causal association between migraine, MA, MO and gut microbiome in reverse MR analyses.

Probiotics administration in preclinical experiments can alleviate the prolongation in the antibiotics-produced migraine-like mice [35]. In clinical studies, probiotic supplementation therapy has demonstrated an effect on the relief of migraine, despite that the result did not always remain consistent [13, 36, 37]. In a randomized controlled trial including a total of 69 female participants with 35 in synbiotics and 34 in placebo groups for 12 weeks, the frequency of attacks and the severity of migraine were dramatically decreased in placebo groups compared with the synbiotics group. The inflammatory biomarkers and gut permeability were decreased [12]. The synbiotics 10 [9] CFU of 12 types of probiotics were included, such as *Bifidobacterium breve*, *Bifidobacterium longum*, and *Bifidobacterium lactis*. A randomized double-blind controlled trial including 40 episodic and 39 chronic migraine patients has also reported that probiotic supplementary might be an effective and beneficial treatment to improve the symptoms of migraine. The *Bifidobacterium* was one of the important components for probiotic supplementary in this study [38]. This evidence suggested that *Bifidobacterium* may play a vital role in the development of migraine, and the *Bifidobacterium* supplement might alleviate the detrimental effects of migraine. In our MR study, we found the causal effects of the dysbiosis of *Bifidobacterium* on the development of migraine and MO. In addition, the causal association still exists after the FDR correction.

The findings of bidirectional interactions between gut and brain in gut-brain axis may be helpful to clarify the underlying mechanisms. Inflammation and neuroimmune regulation in GI could exert an essential influence in the pathological pathway of migraine [39]. In “leaky gut” hypothesis, the inflammatory and immune response are initiated with the increased gut permeability, which would be reinforced after the next secretion of pro-inflammatory factors [40]. It has been reported that proinflammatory factors, such as TNF-α and IL-1β, may be associated with the release of neuro-mediators of pain in the migraine initiation [41–43]. On the other hand, in experimental model of dietary migraine, the pain duration was prolonged because of the disruption of immunopeptidergic network and subsequent dysbiosis of gut microbiota after the application of antibiotic treatment in nitro-glycerin-induced acute migraine-like pain in mice [44] IHowever, the pain prolongation completely disappeared when the effect of TNF-α was blocked through genetic depletion of TNF-α and intra-spinal trigeminal nucleus caudalis injunction of TNF-α antagonist [45]. Therefore, the involvement of up-regulation TNF-α level contributes to the chronicity of migraine-like pain [35]. Short-chain fatty acids (SCFAs) are produced by bacteria in gut, which is a crucial component in the integrity of gut barrier integrity [46, 47]. Apart from affecting gut immunity, SCFAs could reach the CNS via circulation to play a role of neuroprotectiveand anti-inflammatory effects [47]. It can stimulate cell proliferation and differentiation, strengthen the expression of neurotrophins, such as brain-derived neurotrophic factor (BDNF) and glial-derived neurotrophic factor (GDNF), and suppress the synthesis of TNF-α. The supplement of probiotica and the fecal microbiota transplant could robustly reverse microbiota-deprecation-caused migraine and restore the levels of bifidobacterial, indicating the significant impact of dietary factors on the composition and maintenance of the gut microbiota [48].

The dysfunction of gut-brain axis has been investigated in some neurological disorders, including multiple sclerosis [49] and Alzheimer's disease [50]. Our study further provides a strong evidence of the role of the gut-brain axis in migraine. There are some advantages in our study. This MR study first illustrates the role of gut microbiota in the development of migraine. Moreover, the results of our MR study are conducted in multiple migraine datasets and causal effects of the dysbiosis of *Bifidobacterium* on the development of migraine and MO remain robust in both IVW and FDR correction, which suggest *Bifidobacterium* may be a vital therapeutic target for migraine and MO. As a non-invasive approach, the gut microbiome test may be performed to evaluate the risk of migraine, MA, and MO based on specific abundant species in the future, especially for individuals with high-risk factors including hypertension and obesity. The composition of gut microbiome might be affected by diet. As a results, the dysbiosis could be restored using probiotics supplementary interventions. Some studies have suggested the effect of dietary interventions on migraine control [51, 52], such as dietary approaches stop hypertension (DASH) diet and ketogenic diet. The underlying mechanism may be related to the restoration of dysbiosis in gut. The identification of specific bacterial traits can also provide valuable clues for targeted therapeutic approaches. In our MR analyses, a variety of bacterial traits were identified, and the combined benefits of bacterial features can be achieved through fecal transplantation.

Several limitations should be mentioned. GWAS summary-level data are mainly from European participants, which limit the generalizability of our findings. Second, the individual-level association is not obtained due to the unavailable genetic information of the subjects. Third, it is important to acknowledge the impact of low statistical power due to the huge variance in the number of samples between cases and controls, which make us unable to detect the potential causal effects. When interpreting our results, we need to be cautious and fully consider the statistical power although FDR correction to improve the statistical power. Lastly, the results in MR-Egger and weight median are insignificant. Future studies in multi-ancestry and larger sample sizes are needed to verify the conclusions in multiple ancestry with larger sample size.

## Conclusion

Our study explores the possible mechanism of the gut-brain axis in migraine. We identify causal links between the gut microbiome and the development of migraine, MA, and MO. The dysbiosis of *Bifidobacteriaceae* may play an important in the development and prolongation of migraine and its subtypes. Gut microbiome composition may serve as promising biomarkers and therapeutic targets for migraine, MA, and MO. Future research is needed to verify the causal association between gut microbiome and migraine and clarify the specific mechanism.

## Supplementary Information

**Additional file 1: Table S1.** Single-nucleotide polymorphisms used as instrumental variables at the threshold of *P*<1e-5 in MR analysis. **Table S2.** Single-nucleotide polymorphisms used as instrumental variables at the threshold of *P*<1e-8 in IHGC datasets in MR analysis. **Table S3.** Single-nucleotide polymorphisms used as instrumental variables at the threshold of *P*<1e-8 for FinnGen datasets in MR analysis. **Table S4.** Single-nucleotide polymorphisms used as instrumental variables for migraine at the threshold of *P*<1e-5 in IHGC datasets in reverse MR analysis. **Table S5.** Single-nucleotide polymorphisms used as instrumental variables for MA at the threshold of *P*<1e-5 in IHGC datasets in reverse MR analysis. **Table S6.** Single-nucleotide polymorphisms used as instrumental variables for MO at the threshold of *P*<1e-5 in IHGC datasets in reverse MR analysis. **Table S7.** Single-nucleotide polymorphisms used as instrumental variables for migraine at the threshold of *P*<1e-5 in FinnGen datasets in reverse MR analysis. **Table S8.** Single-nucleotide polymorphisms used as instrumental variables for MA at the threshold of *P*<1e-5 in FinnGen datasets in reverse MR analysis. **Table S9.** Single-nucleotide polymorphisms used as instrumental variables for MO at the threshold of *P*<1e-5 in FinnGen datasets in reverse MR analysis. **Table S10.** Single-nucleotide polymorphisms used as instrumental variables for mmigraine at the threshold of *P*<1e-8 in IHGC datasets in reverse MR analysis. **Table S11.** Single-nucleotide polymorphisms used as instrumental variables for MO at the threshold of *P*<1e-8 in IHGC datasets in reverse MR analysis. **Table S12.** MR results of causal links between gut microbiome and migraine at the threshold of *P*<1e-5 in the IHGC GWAS datasets in MR analysis. **Table S13.** MR results of causal links between gut microbiome and migraine with aura at the threshold of *P*<1e-5 with aura in the IHGC GWAS datasets in MR analysis. **Table S14.** MR results of causal links between gut microbiome and migraine without aura at the threshold of *P*<1e-5 in the IHGC GWAS datasets in MR analysis. **Table S15. **The MR-Egger and Cochran’s *Q *test of gut microbiome on migraine, migraine with aura and migraine without aura at the threshold of *P*<1e-5 in the IHGC GWAS datasets in MR analysis. **Table S16. **Positive MR results of causal links between gut microbiome and migraine, migraine with aura and migraine without aura at the threshold of *P*<1e-5 in the FinnGen GWAS datasets in MR analysis. **Table S17.** MR results of causal links between gut microbiome and migraine at the threshold of *P*<1e-5 in the FinnGen GWAS datasets in MR analysis. **Table S18.** MR results of causal links between gut microbiome and MA with aura at the threshold of *P*<1e-5 in the FinnGen GWAS datasets in MR analysis. **Table S19.** MR results of causal links between gut microbiome and MO without aura at the threshold of *P*<1e-5 in the FinnGen GWAS datasets in MR analysis. **Table S20. **The MR-Egger and Cochran’s *Q *test of gut microbiome on migraine, migraine with aura and migraine without aura at the threshold of *P*<1e-5 in the FinnGen GWAS datasets in MR analysis. **Table S21. **MR results of causal links between gut microbiome and migraine, migraine with aura and migraine without aura in the IHGC GWAS datasets at the threshold of *P*<1e-8 in MR analysis. **Table S22. **MR results of causal links between gut microbiome and migraine, migraine with aura and migraine without aura in the FinnGen GWAS datasets at the threshold of *P*<1e-8 in MR analysis. **Table S23. **The MR-Egger and Cochran’s *Q *test of migraine, migraine with aura and migraine without aura on gut microbiome in the IHGC GWAS datasets at the threshold of *P*<1e-5 in reverse MR analyses. **Table S24. **The MR-Egger and Cochran’s *Q *test of migraine, migraine with aura and migraine without aura on gut microbiome in the FinnGen GWAS datasets at the threshold of *P*<1e-5 in reverse MR analyses. **Table S25. **MR results of causal links between migraine, migraine with aura and migraine without aura on gut microbiome at the threshold of *P*<1e-8 in the IHGC GWAS datasets in the reverse MR analyses. **Table S26. **MR results of causal links between migraine, migraine with aura and migraine without aura on gut microbiome at the threshold of *P*<1e-8 in the FinnGen GWAS datasets in the reverse MR analyses. **Figure S1.** The leave-one-out analyses of bacterial traits for migraine at the threshold of *P*<1e-5 in IHGC datasets in MR analyses. **Figure S2.** The leave-one-out analyses of bacterial traits for MA at the threshold of *P*<1e-5 in IHGC datasets in MR analyses. **Figure S3.** The leave-one-out analyses of bacterial traits for MO at the threshold of *P*<1e-5 in IHGC datasets in MR analyses. **Figure S4.** The common bacterial traits among migraine, MA, and MO at the threshold of *P*<1e-5 in IHGC datasets in MR analyses. **Figure S5.** Causal effect of the gut microbiome on migraine, MA, MO at the threshold of *P*<1e-5 in FinnGen datasets based on MR analyses in MR analyses. **Figure S6.** Causal effect estimates of the gut microbiome on migraine at the threshold of *P*<1e-5 in FinnGen datasets in MR analyses. **Figure S7. **Causal effect estimates of the gut microbiome on MA at the threshold of *P*<1e-5 in FinnGen datasets in MR analyses. **Figure S8. **Causal effect estimates of the gut microbiome on MO at the threshold of *P*<1e-5 in FinnGen datasets in MR analyses. **Figure S9.** The leave-one-out analyses of bacterial traits for migraine at the threshold of *P*<1e-5 in FinnGen datasets in MR analyses. **Figure S10.** The leave-one-out analyses of bacterial traits for MA at the threshold of *P*<1e-5 in IHGC datasets in MR analyses. **Figure S11.** The leave-one-out analyses of bacterial traits for MO at the threshold of *P*<1e-5 in IHGC datasets in MR analyses. **Figure S12.** The common bacterial traits among migraine, MA, and MO at the threshold of *P*<1e-5 in FinnGen datasets in MR analyses. **Figure S13.** Causal effect estimates of the migraine on gut microbiome at the threshold of *P*<1e-5 in IHGC datasets in reverse MR analyses. **Figure S14.** Causal effect estimates of the MA on gut microbiome at the threshold of *P*<1e-5 in IHGC datasets in reverse MR analyses.** Figure S15.** Causal effect estimates of the MO on gut microbiome at the threshold of *P*<1e-5 in IHGC datasets in reverse MR analyses. **Figure S16.** The leave-one-out analyses of migraine on gut microbiome at the threshold of *P*<1e-5 in IHGC datasets in reverse MR analyses. **Figure S17.** The leave-one-out analyses of MA on gut microbiome at the threshold of *P*<1e-5 in IHGC datasets in reverse MR analyses. **Figure S18.** The leave-one-out analyses of MO on gut microbiome at the threshold of *P*<1e-5 in IHGC datasets in reverse MR analyses. **Figure S19.** Causal effect estimates of the migraine on gut microbiome at the threshold of *P*<1e-5 in FinnGen datasets in reverse MR analyses. **Figure S20.** Causal effect estimates of MA on gut microbiome at the threshold of *P*<1e-5 in FinnGen datasets in reverse MR analyses. **Figure S21.** Causal effect estimates of MO on gut microbiome at the threshold of *P*<1e-5 in FinnGen datasets in reverse MR analyses. **Figure S22.** The leave-one-out analyses of migraine on gut microbiome at the threshold of *P*<1e-5 in FinnGen datasets in reverse MR analyses. **Figure S23.** The leave-one-out analyses of MA on gut microbiome at the threshold of *P*<1e-5 in FinnGen datasets in reverse MR analyses. **Figure S24.** The leave-one-out analyses of MO on gut microbiome at the threshold of *P*<1e-5 in FinnGen datasets in reverse MR analyses. **Figure S25.** Causal effect estimates of migraine on gut microbiome at the threshold of *P*<1e-8 in IHGC datasets in reverse MR analyses. **Figure S26.** Causal effect estimates of MO on gut microbiome at the threshold of *P*<1e-8 in IHGC datasets in reverse MR analyses. **Figure S27.** The leave-one-out analyses of migraine on gut microbiome at the threshold of *P*<1e-8 in IHGC datasets in reverse MR analyses. **Figure S28.** The leave-one-out analyses of MO on gut microbiome at the threshold of *P*<1e-8 in IHGC datasets in reverse MR analyses.

## Data Availability

The original contributions presented in the study are included in the article/Supplementary Material, further inquiries can be directed to the corresponding author.
